# The challenges of implementing national policies to contain antibiotic resistance in Swedish healthcare—A qualitative study of perceptions among healthcare professionals

**DOI:** 10.1371/journal.pone.0233236

**Published:** 2020-05-20

**Authors:** Marta Röing, Ingeborg Björkman, Jaran Eriksen, Cecilia Stålsby Lundborg

**Affiliations:** 1 Department of Public Health and Caring Sciences, Health Services Research, Uppsala University, Uppsala, Sweden; 2 Department of Global Public Health–Health Systems and Policy (HSP): Improving the Use of Medicines, Karolinska Institutet, Stockholm, Sweden; 3 Unit for Infectious Diseases, Venhälsan, Södersjukhuset, Stockholm, Sweden; Newcastle University, UNITED KINGDOM

## Abstract

**Objective:**

To explore and describe how healthcare policymakers and healthcare practitioners from different levels of Swedish healthcare perceived the everyday practice of putting national policies to contain antibiotic resistance into effect.

**Method:**

A strategic sample of four healthcare policymakers, three healthcare practitioners working in hospital care, and six working in primary care were recruited and interviewed in person. A manifest and latent content analysis was carried out on the transcribed interview data.

**Results:**

Analysis revealed how the everyday practice of containing antibiotic resistance in different healthcare levels could be perceived as successful, difficult, or a dilemma. The informants’ perceptions are presented in three categories which describe first; informants’ perceptions of antibiotic use and antibiotic resistance in Sweden; secondly, informants’ perceptions of definable efforts in containing antibiotic resistance in Sweden, most notably responsible use of antibiotics, prevention of infection, improving public knowledge of antibiotic resistance, and international collaboration; and thirdly, informants’ perceptions of problem areas in containing antibiotic resistance in Sweden, such as behavior, attitudes and knowledge among healthcare practitioners and the public, work environment, and resources. Reflection on the underlying meaning of these perceptions led to identification of the latent theme, labelled “A sense of relative success, and many challenges yet to overcome”.

**Conclusion:**

This study has given in-depth insight into how a group of healthcare policymakers and practitioners perceived the everyday practice of containing antibiotic resistance, and revealed their perceptions of successful efforts to combat antibiotic resistance so far. It has identified problem areas in different healthcare levels, bringing to light challenges yet to overcome, and areas to focus on in future policies, most notably more emphasis on attitude and behavior change, and increasing awareness of antibiotic resistance among both healthcare practitioners and patients.

## Introduction

Antibiotic resistance (AR) is recognized as a significant threat to global health in modern times [1]. Although the importance of government level strategies and policies to combat this threat cannot be denied, evaluating the extent to which they are successfully implemented and contribute to these efforts is difficult [2].

Efforts to combat antibiotic resistance are being driven by global organizations, such as the World Health Organization (WHO), World Organization for Animal Health (OIE), United Nations Food and Agriculture Organization (FAO), as well as health communities in Europe, North America, and Australia [3]. In recent years, WHO, OIE, and FAO have adopted a ‘One Health’ approach in work to contain AR. This approach is broadly defined as “the collaborative effort of multiple disciplines- working locally, nationally and globally–to attain optimal health for people, animals and our environment” [3].

In Sweden, systematic initiatives to contain antibiotic resistance beginning in the mid-1990’s have contributed to the present favorable situation of having limited antibiotic resistance and low antibiotic consumption [4]. One notable initiative has been the creation in 1995 of the Swedish strategic program against antibiotic resistance (Strama). It began as a voluntary network of professional organizations and multi-professional groups with an overall aim of safeguarding the efficiency of antibiotic treatment in humans and animals. In 2006 it was officially assigned the task of facilitating coordination of work against antibiotic resistance by the government [4, 5].

Strategies to combat antibiotic resistance adopted by the Swedish government in 2016 included “continuous collection of data on the occurrence of resistant bacteria and use of antibiotics”, “continuous strong preventive measure to minimize the spread of multi-resistant bacteria”, “responsible use of antibiotics, ‘”improved awareness and understanding in society about antibiotic resistance”, “clear systems and structures for collaboration” [6].

Policies are ways of expressing ongoing strategies, the formal written plans, rules and guidelines for accomplishing governmental goals [7]. In accordance with official strategies mentioned above, the Swedish government expected relevant staff to have “knowledge about antibiotic resistance, the spread of infectious diseases, compliance with basic hygiene routines and other preventive measures, knowledge about the seriousness and complexity of the issue from a global perspective”. The government expected “Strama to lead national work”, and “people working in health care and social services, dental care, with animals and with food production to have good knowledge of the One Health concept” [6].

Policy implementation depends on frontline providers to carry out the daily practice of putting plans into effect [8]. For a policy to succeed, providers must adhere to policy rules and guidelines. Healthcare is complex, with multiple sectors and organizational levels, and is delivered by practitioners with different roles, responsibilities, and training. As such, adhering to policy guidelines may not be straightforward, and potentially useful policies cannot be implemented the way they were intended [9].

Against this background, our aim was to explore and describe how healthcare policymakers and healthcare providers at different levels of Swedish healthcare perceived the everyday practice of putting national policies to contain antibiotic resistance into effect. Inquiring into such perceptions can lead to a deeper understanding of daily antibiotic resistance work from the perspective of these individuals, how they look upon antibiotic resistance, and problems they may face, if any. This knowledge can in turn indicate directions for future strategies and policies to contain antibiotic resistance.

## Method

This study is part of ABRCARRO (A One Health Systems and Policy Approach to Antibiotic Resistance Containment: Coordination, Accountability, Resourcing, Regulation and Ownership)—an international project which aims to explore and describe how national action plans on antibiotic resistance were developed, implemented, monitored and evaluated in Sweden, South Africa and Swaziland. The project includes interviews with different categories of stakeholders at government level, for example policymakers, and professionals in human, animal, and environment/agriculture sectors, as well as policy document analyses. This study focuses on the human sector of Swedish healthcare.

### Design

A qualitative research approach with an inductive and descriptive design was chosen. An inductive approach involves drawing codes, categories or themes directly from the data, and is useful when knowledge about a phenomenon is limited [10].

### Informants

A strategic sample of 13 informants was recruited, and included four from the policymaking level, three from hospital care, and six from primary care (PC) (Table 1). Informants at each level contributed to antibiotic resistance work in different ways, and exploring these levels allowed examination of and insight into these perspectives.

**Table 1:** Informants.

Healthcare level	Informants
Four professionals at policymaking level	One analyst working at the Public Health Agency of Sweden.
	One physician working at Public Health Agency of Sweden.
	One physician and Chairperson of Strama primary care.
	One dentist specialist and member of Strama for dentists.
Three practitioners in hospital care	Physician specialist in infectious diseases and member of Strama.
	Physician specialist in paediatrics.
	Physician specialist in orthopaedics.
Six Practitioners in primary care	Three physician specialists in family medicine, two from a primary care clinic with low prescription of antibiotics and one from a clinic with high prescription of antibiotic. One was also manager of the clinic.
	Three registered nurses, one from a primary care clinic with high prescription of antibiotics, one responsible for health care issues in municipal elderly care, and one working at the national telephone healthcare advice services.

### Data collection

Thirteen interviews were conducted by author IB between January and September 2018. The interviews lasted from 31 to 88 minutes (average approximately 50 minutes). All possible informants had been contacted via email, informed of the purpose of the study, and asked to participate. One declined due to time constraints, and as a result another informant from the same healthcare level was contacted by email. After not responding to this email, this informant was contacted again, this time personally, asked to participate, and agreed. Specific interview guides were used for the policymaking and healthcare practitioner informants. These guides were based on an interview guide previously used by the research group when studying perceptions of antibiotic use and antibiotic resistance (S 1), and where some questions had been adjusted to focus on the One Health approach (Table 2). Interview questions were followed by probing questions, for example, asking why, how, or requesting informants to expand on their responses, or give more detail. The interviews were then carried out at a place chosen by the informants. All interviews were audio-recorded and transcribed verbatim. Before analysis the interviews were listened through and transcripts checked and corrected by author IB.

**Table 2:** Main interview questions for healthcare policymaking and healthcare practitioner informants.

1. What does antibiotic resistance mean to you?
2. How do you look upon your role in working to contain antibiotic resistance?
3. How do you look upon possibilities of limiting/preventing emergence and spread of antibiotic resistance?
4. What do you think are the main causes of antibiotic resistance?
5. How do you think antibiotic resistance spreads?
6. How do you look upon the use of antibiotics in humans, animals, or any other areas?
7. Have you heard of the concept of ‘One Health’?
8. Do you have any comments to add?

### Data analysis

At start authors MR and IB analysed one (the same) interview separately. This involved reading the interview, marking meaning units, and grouping them under preliminary categories separately. The authors then met, and reached consensus on an initial coding scheme consisting of 25 preliminary categories. Author MR then proceeded with a latent qualitative content analysis inspired by Graneheim and Lundman [11]. This meant focusing first on the manifest content of the interview texts, (the obvious, visible components), with no theories or predefinitions, and then interpreting the latent content (the underlying meaning of the text) [11]. The analytic process began by reading transcripts of each interview individually, to get a good grasp of the whole, and then proceeded according to the steps in Table 3. The final analysis was presented to author IB for discussion, and her judgment as to whether it was reasonable based on what had been expressed in the interviews. The remaining authors were then invited to review and discuss the data as analysed. No changes were made after their reviews.

**Table 3:** Steps in process of analysis.

Steps included in the analysis process	
1	Meaning units were identified in all the interviews by MR, one interview at a time, and sorted under the 25 preliminary categories agreed upon with author IB.
2	Preliminary categories were grouped and assigned descriptive codes according to shared content.
3	Preliminary categories were condensed or merged into 10 subcategories.
4	Subcategories were merged into three categories based on research aim.
5	Reflection on the latent content of the interview texts led to identification of a theme, which in this study gave insight into the underlying meaning of work to contain antibiotic resistance for informants.

### Ethical considerations

Ethical approval was sought from the Regional Ethics Board, Stockholm (Reg number: 2017/1999-31 According to an advisory opinion from the Board, there were no ethical objections to the study. The informants were guaranteed confidentiality, and informed that participation was voluntary, and that they were free to withdraw from the study at any time. Written consent was obtained from all informants.

## Findings

The informant’s perceptions are presented in three categories labeled: (A) Perceptions of antibiotic use and antibiotic resistance in Sweden; (B) Perceptions of definable efforts in containing antibiotic resistance in Sweden; (C) Perceptions of problem areas in containing antibiotic resistance in Sweden. Reflection on the underlying meaning in the perceptions led to identification of the latent theme, which was labelled ‘A sense of relative success, and many challenges yet to overcome’. The theme and three categories, together with their subcategories, are presented in Table 4. A description of each category illustrated by quotations from the interviews follows. All the informants have contributed to the quotations, which are identified according to informant professional group and healthcare level, and interview number.

**Table 4:** Theme, categories and subcategories of how a group of healthcare policymakers and practitioners perceived the everyday practice of putting national policies to contain antibiotic resistance into effect.

Subcategories	Categories (Manifest content)	Theme (Latent content)
1. Antibiotic use	A. Perceptions of antibiotic use and antibiotic resistance in Sweden	
2. Antibiotic resistance		
1. Responsible use of antibiotics	B. Perceptions of definable efforts to contain antibiotic resistance in Sweden	A sense of relative success, and many challenges yet to overcome
2. Prevention of infection		
3. Behavior, attitudes and knowledge—patients/public		
4. Collaboration		

1. Behavior attitudes and knowledge–healthcare practitioners	C. Perceptions of problem areas in containing antibiotic resistance in Sweden	
2. Behavior, attitudes and knowledge–patients/public		
3. Work environment		
4. Resources		

### A. Perceptions of antibiotic use and antibiotic resistance in Sweden

This category gives a general description of how the informants looked upon antibiotic use and antibiotic resistance in Sweden.

#### 1. Antibiotic use

All informants shared the opinion that the use of antibiotics in Sweden was much better now than 20, 30 years ago, and that Sweden is one of the furthest in restrictive antibiotic use compared to other countries. *“It [antibiotic use*] *has become lot better if you compare to how it was in the 80’s and 90’s*. *We’ve seen that the use of antibiotics has been halved*, *at least for children*, *compared to the 90’s*. *And we haven’t seen any increase in complications*…*that patients suffer in any way*. *So antibiotic use in Sweden is pretty good*, *in any case compared to other countries” (PC Physician H2)*.

*“We are generally careful with antibiotics …*.*we don’t prescribe them if they are not necessary… we start with simple measures for fever*, *colds*, *and so on*, *so not using antibiotics unnecessarily is always in the back of our minds*.*” (PC physician H10)* This quotation is an example of how one informant understood the need for healthcare practitioners to be restrictive in prescribing antibiotics and the need for people to refrain from using antibiotics, if possible. “*Every antibiotic cure upsets the bacterial balance in the stomach even more…and can result in resistant bacteria attaching themselves …so every antibiotic cure can disturb intestinal flora for almost a year…you can say…and then it is easier during that year to be infected by resistant bacteria*…*so that’s why you really have to evaluate every individual antibiotic cure…is it really necessary*?*” (Hospital specialist physician H6)*.

Even so, there was an opinion that antibiotics were still being used unnecessarily in Sweden.”*Still*, *you can wonder if it really is correct that one of three patients are given a dose of antibiotics*, *at least once during their hospital stay*. *So…antibiotics are being overused*, *even in hospitals*.*” (Hospital specialist physician H6)*.

Among healthcare practitioner informants there was a general perception that antibiotics were being misused in other countries. Their opinions in this matter were based on personal experience and observations when working abroad, or from scientific and specialized press, and media coverage. *“I’ve worked in Ethiopia for example*, *where amoxicillin was doled out …to children*. *But there I thought the same way*, *play it safe*, *there you have all the more reason to do so …children are undernourished and sick…but yes*, *generally speaking I believe many received antibiotics without needing it” (Hospital specialist physician H9)*.

Informants’ awareness of the use of antibiotics in animal food production was limited, and also based on media coverage. Very few informants were aware of, or had much knowledge of environmental effects of antibiotics and other drugs.

#### 2. Antibiotic resistance

Policymaking informants had the perception that people felt ‘relatively safe’ in Sweden regarding antibiotic resistance. There was a belief that food in Sweden was produced under good conditions, and therefore was there no need to worry about resistant bacteria or antibiotics in food. Instead, there was a belief that antibiotic resistance was imported into Sweden, via travel, picking up bacteria during hospital stays for cheaper medical care in other countries, eating contaminated food, abroad or at home. *“I think a lot about all the plastic surgery operations being carried out abroad…*.*where they are so much cheaper…so people travel abroad*, *have a lot of operations*, *and then come back with MRSA bacteria*. *And maybe they don’t say anything when they visit a hospital*, *which spreads infection even more” (PC nurse H8)*.

There was an impression among the informants that antibiotic resistance was still a small problem in primary care. If and when it was suspected, patients were treated according to specific routines, or referred to hospitals. *“An ordinary doctor comes upon it [antibiotic resistance*] *two*, *three*, *four times a year*, *or something like that*…*a urinary tract infection that doesn’t get better*, *and results in pyelonephritis*.*” (PC physician H2)* Antibiotic resistance was also not a real issue in dental care. *“It’s not as if…*.*resistant bacteria cause oral infections*, *that’s not the way it is…*.*no*.*” (Policymaker H12)* However, according to policymaking informants, resistant bacteria have been involved in outbreaks of infection in hospitals. *“Resistant bacteria*, *we have had outbreaks…we have had big ones involving many hospitals…Vancomycin resistant enterococci for example*. *And then infection spread in neonatal wards with serious consequences*. *So the topic is highly relevant in Sweden as well” (Policymaker H4)*.

All the informants were aware of the serious consequences of antibiotic resistance, that, in the end, it might not be possible to treat infections with antibiotics at all, and people could die. Policymaking informants had the most extensive knowledge of antibiotic resistance. They felt that people did not really understand the paralyzing breadth of antibiotic resistance, and the death and suffering it could lead to. One policymaker noted that mankind did not react to slow scenarios, where it was possible to adapt slowly, and compared the threat of antibiotic resistance to climate change, something not yet experienced as a direct threat. Antibiotic resistance was a ticking bomb, a threat today, and a potentially greater threat in the future. And the future, according to the following policymaker, was very uncertain. *“Imagine a mountain side with water running into a lake containing all the antibiotic resistance*. *We [Sweden*] *are a tiny stream*, *and then you have a giant rapid*, *a glacial rapid*, *coming from the rest of the world*, *Of course it doesn’t matter what we do*.. *it won’t be noticeable*…*our efforts almost make no difference in the overall context” (Policymaker H*.*7)*.

### B. Perceptions of definable efforts in containing AR in Sweden

This category presents informants’ spontaneous examples of efforts where they felt they had been successful in their work to contain antibiotic resistance.

#### 1. Responsible use of antibiotics

One policymaking informant described how work to decrease the use of antibiotics had first focused on primary care, since antibiotics appeared to be overused in many PC clinics. And it was perceived that these efforts had been successful. All the informants were very aware of Strama’s role in facilitating responsible antibiotic use and increasing awareness of antibiotic resistance in primary care. Examples included how PC clinics were regularly visited by healthcare professionals working with Strama, who informed about antibiotics and infection treatment. *“These [Strama*] *doctors have been very good at coming out and informing and putting pressure on our doctors…*.*like…to reduce… and make the right diagnosis …*.*most importantly the right diagnosis…*.*certain diagnoses do not need antibiotics” (PC nurse H11)*.

Recommendations for correct antibiotic use in dentistry had been revised in 2014, in collaboration with Strama; telenurses used telephone advice guidelines to advise callers, which also followed Strama guidelines on antibiotic use; Strama regularly followed up antibiotic use in primary care and public health dental clinics; hospitals received data on their total antibiotic use from Strama every 3 months, but how they used this information varied. *“In spite of all the alarm reports*, *I believe*, *and if you ask my colleagues*, *that the use of antibiotics in Sweden is going in the right direction That is what I believe and how I feel*, *and my colleagues feel the same way*. *On the other hand I don’t know how that is affecting antibiotic resistance*, *but we are much more rational now than 15 years ago” (PC physician H1)*.

There was a general opinion about primary care, that it was easier to be more restrictive when prescribing antibiotics, since infections in primary care require a completely different spectrum of antibiotics compared to infections treated in hospitals. It was a question of choosing where to draw the line, prescribe less antibiotics, only when necessary, and less broad spectrum antibiotics. A policymaking informant had similar thoughts about dentists. “*Patients seek acute dental treatment*, *for swelling*, *but most of all for pain…toothache*. *And antibiotics are not a way to treat toothache…so many times it is enough with local treatment combined with pain relief” (Policymaker H12)*.

#### 2. Prevention of infection

*“The chances of being infected with resistant bacteria in the subway is almost zero…*.*there is little risk of being infected in outpatient clinics*, *but it can be a reality in a hospital*.*” (PC physician H2)* According to one hospital specialist informant, strategic efforts on part of hospital management had led to better hygiene routines in hospitals, which were followed a lot better now compared to 15 years ago.

Primary care had strict routines regarding clothes and hand hygiene, to prevent the spread of infection, not just resistant bacteria. Hygiene routines in dental clinics followed national guidelines. In nursing care of the elderly a special hygiene nurse had hygiene rounds to control how staff followed basal hygiene routines. Newly hired and temporary staff was required to learn basal hygiene routines, and even sign a document where they agreed to work according to these rules.”*Then we have a hygiene nurse in charge of educating personnel*…*different personnel categories*, *from assistant nurses*, *ward aids*, *paramedics*, *nurses*, *to department heads are expected to register…*. *and who also is responsible for hygiene rounds*, *to check up on how we work with basicl hygiene routines (PC nurse H5)*.

All PC clinics had somebody responsible for infection control. One PC physician informant was part of a Strama network comprised of other doctors, disease control specialists, and nurses from other PC clinics in the region. Their network disease control meetings covered many areas, for example chlamydia, HIV, hygiene, as well as antibiotic resistance. One PC nurse informant working in municipal elderly care described her role in being responsible for keeping statistics on infections in elderly care, and how she was always informed if new patients coming from hospitals were carrying resistant bacteria.

#### 3. Behavior, attitudes and knowledge–patients/public

One PC physician informant felt it was important to make patients understand why antibiotics were not always necessary. A PC nurse informant, on the other hand, thought that doctors were often stressed, and had little time to inform patients properly. According to this informant, patients received better information from nurses than doctors, who were often stressed, with little time to inform patients properly.*”You try to explain about resistance…I say [to the patient*] *‘ imagine if you really get sick in the future…and need antibiotics…and they don’t help…how would you feel*?*’*… *and then I talk a lot about intestinal flora…*.*I usually say you may win a few days…*.*but think about how you are destroying your stomach and intestines…*..*it takes many months to build up your normal intestinal flora again” (PC nurse H11)*.

All the informants commented on the media’s role in changing the publics’ expectations regarding antibiotics. *“I think the media has played a very important role…*.*describing the problem [antibiotic resistance*] *… reporting successes and statistics and such…it has been very engaged and …*.*has understood the importance*. *There are many medical journalists who have understood the importance through the years and this has resulted in general reporters joining in*. *That’s how it sunk in*.*…these newspapers are read by decision makers*, *doctors*, *veterinarians*, *and the general public*.*” (Policymaker H3)* People were beginning to understand the negative effects for self and society, many even were trying to avoid taking antibiotics, if possible. People who insisted on antibiotic treatment still existed, but were fewer in number, and often badly informed.

#### 4. Collaboration

Policymaking informants described their collaboration with animal and environment health sectors in Sweden, and their involvement in global efforts to contain antibiotic resistance. Examples included representing Sweden in projects together with other EU countries, such as monitoring antibiotic resistance and healthcare associated infections, and regular meetings to create national action plans for antibiotic resistance work. *“They [EU countries*] *are very interested…it’s not as if they just sit there because they have to…but they are very engaged in what needs to be done and come with suggestions …so it is very promising*.*” (Policymaker H4)* And, there was a perception that success of Swedish efforts in antibiotic resistance work so far had resulted in an interest in Sweden as a role model.

The policymaking informants also had a good understanding of the realities of global collaboration, how antibiotic resistance work was affected if countries had to deal with lack of water, climate change, natural disasters, and limited resources. For example, Bric countries (Brazil, Russia, India, China) wanted the same healthcare outcomes as industrialized countries, but did not have economical or hygienic conditions, and compensated by using a lot of AB in healthcare and food production. It was very hard to stop the actions of these countries. “*People want to keep their eyes closed to the problem of antibiotic resistance*, *there are too many economical consequences” (Policymaker H7)*.

### C. Problem areas in containing antibiotic resistance in Sweden

This category gives insight into informants’ perceptions of problems they faced in their work to contain antibiotic resistance.

#### 1. Behavior, attitudes and knowledge–healthcare professionals

In primary care, as noted by PC informants, there were differences in how physicians trained abroad looked upon antibiotics compared to physicians trained in Sweden. There was an impression that some foreign physicians in Sweden still seemed to work according to the antibiotic culture in their homeland, that of treating common infections with antibiotics. As a result, antibiotics appeared to be used a lot in areas with high concentration of immigrants, and in PC clinics with many foreign physicians. *“Higher antibiotic prescribing in some primary care clinics does not always mean sicker patients*, *but maybe a different culture”* (*PC physician H10)*.

Decreasing the use of antibiotics in hospitals was not always straightforward. For example, some specialist physicians felt uncomfortable about treating seriously ill hospital patients with narrow spectrum antibiotics. In pediatrics, the practice was to start with broad spectrum antibiotics, before resistance testing, since pediatric specialists could fear the serious consequences of infection in small children.”*So the difficulty*, *I believe*, *is when you stand there at night…from my perspective…*.*in the darkness of night and do not really know how sick a child is…*.*so you choose maybe a broader antibiotic …or antibiotics when you don’t really need them*.*” (Hospital specialist physician H9)* As noted by hospital specialist physician informants, antibiotics could be overused to some extent in orthopedic hospital care, as it could be hard to diagnose if or when an infection did not require antibiotics. Furthermore, they noted that regular follow-ups on antibiotic use were not always carried out in orthopedic, or pediatric hospital clinics.

Policymaking informants had the opinion that the mindset of ‘it never hurts to prescribe antibiotics’ needed to be changed, and questioning if antibiotics were necessary in treatment planning should be put into practice in hospitals. They realized how difficult this could be for some younger doctors.”*Sometimes you can be forced to prescribe something only because your superior thinks you should and because there are many doctors involved in the process*.*” (Policymaker H7)* The policymaking informants were also aware that some doctors were not interested in changing their ways.”*Thinking Sweden is good when it comes to antibiotic resistance work can lead to arrogance among healthcare professionals*, *that we are done*, *and do not need to bother anymore” (Policymaker H3)*.

Most of the informants had limited knowledge of antibiotic work internationally, in animal food production, and in the environment. Very few were aware of the One Health concept. One PC physician informant thought that One Health was not important to physicians in local PC clinics, and that One Health should work from a higher level, coordinating animal and human sectors, and exerting pressure internationally. This informant would also have liked to see real evidence that antibiotics in animal food-production contribute to antibiotic resistance in humans.

#### 2. Behavior, attitudes and knowledge–patients/public

One policymaking informant observed that vaccinations to prevent viral infections that have bacterial consequences could result in less antibiotic use, and, although rare, it was unfortunate that some children were not being vaccinated today, since these children could end up in emergency pediatric clinics. *“Many parents are very convinced and well read*, *but base their opinions on wrong sources” (Hospital specialist physician H9)*.

PC physician informants were aware that some patients travelled abroad, stocked up on antibiotics to sell to friends when home in Sweden, or took only half their antibiotic cure, to save for the next times. They understood that many of these patients did not believe they would get better without antibiotics. Patients could also demand antibiotic prescriptions from their dentists before going abroad. One PC nurse informant working in telephone advice services described feelings of anger when patients called in and admitted they had bought antibiotics abroad. It was difficult to convince some people to begin with over-the-counter medicines, and try to endure, especially if they were parents of small children with fever. This informant had observed people’s concerns about antibiotics, which may be why they called Sweden’s national telephone advice services. “*They say … doctors don’t have the time to listen to us*, *or they didn’t listen*, *or even inspect my throat*, *we just got a prescription” (PC nurse H8)*.

#### 3. Work environment

Informants in both hospital and primary care remarked on their stressful work environment, there was just not enough time to convince and inform patients about antibiotic use and antibiotic resistance. Physician informants in both primary care and hospitals said they ended up prescribing antibiotics when stressed and felt the need to act. Furthermore, stress, short hospital stays, lack of beds could result in choosing broad spectrum antibiotics instead of narrow spectrum antibiotics, and which also increased the risk of infection spread in hospitals. And, a very high turnover of hospital staff made antibiotic resistance work, and education of staff difficult. There was a shared opinion that hospital management should be more open and sensitive to the problem of antibiotic resistance, and should take larger responsibility for antibiotic use and antibiotic resistance issues. “*If you are going to have the opportunity to admit a patient*, *to wait and see what happens before setting in antibiotics*, *you need an understanding environment*, *colleagues*, *a bed*,…*and you need more time” (Hospital physician specialist H6)*.

#### 4. Resources

Resources seemed to be an area of concern for most informants, including even some policymaking informants. It was hard to know if there were enough resources for antibiotic resistance work, funding of campaigns, research, etc.”*It is quite fascinating that everybody says this [antibiotic resistance*] *is one of the biggest problems we have*, *a future problem from a global perspective*. *Still there is no money*. *The Swedish Research Council abstained from targeting money for antibiotic resistance research…*.*it was targeted for infections*, *yes*, *and now we have stronger focus on virus research and such and I don’t think there is much left over for antibiotic resistance research*.*” (Policymaker H7)* International cooperation, according to another policymaking informant, was one way that Sweden, as a role model, could spread knowledge about AR work, and limited resources made it hard to respond to international demands for this knowledge.

## Discussion

In summary, insight into perspectives, and from different healthcare levels, has revealed how the daily practice of containing antibiotic resistance could be perceived as successful, difficult, and/or a dilemma. We follow with a discussion of our findings against this background.

The informants’ perceptions of successful efforts in responsible use of antibiotics, prevention of infection, improving public knowledge of antibiotic resistance, and international collaboration are in accordance with Swedish official strategies presented in our introduction, suggesting that policy rules and guidelines were being adhered to in these areas. Relevant healthcare providers also appeared to have “knowledge about antibiotic resistance, the spread of infectious diseases, compliance with basic hygiene routines and other preventive measures, knowledge about the seriousness and complexity of the issue from a global perspective,” as expected by the Swedish government [6].

The Swedish context of this study may have contributed to informants’ perceptions of success in these areas. The use of antibiotics differs across Europe, with the lowest level of antibiotic use in Northern countries, and highest in Mediterranean countries [12]. Many factors, for example doctor, patient characteristics, the doctor-patient relationship, contribute to these differences [13]. Cultural factors play an important role in public attitudes towards antibiotic use, how people behave during illness, use antibiotics or self-medicate [13].

Implementation of health strategies and policies is also influenced by context [14]. National cultural dimensions, according to Borg, are formed from care values in society, which have evolved over many years [12]. The possibility does exist that shared values in Sweden have contributed to the success of many efforts to contain antibiotic resistance so far. Countries differ in how they govern, as well as how they think and talk about health [13, 15]. In Sweden, most health problems have been explained by a combination of causes associated with structural factors in society, as well as individual behavior [15]. Public health in Sweden is looked upon as ‘society’s responsibility’[16]. Early public health initiatives in Sweden focused on influencing ‘the attitudes and values of individuals, so that they chose by themselves to live a healthier and less risky life” [15]. Discussions on changes were then argued as a way to help those who would be harmed by unhealthy behavior, as an act of solidarity. The media did play a large role in informing the public about the risks of antibiotic resistance, but it was the Swedish people who accepted and took on this responsibility, and ‘did their part’ in trying to reduce their use of AB. This sense of solidarity, in contrast to individual centering, seemed to be evident among informants on the primary care level as noted in their satisfaction when describing successful efforts in working towards decreasing antibiotic use in Sweden, and how these feelings appeared to be marred by frustration with physicians who did not practice restrictive antibiotic use, or patients who insisted on antibiotics, or did not comply with given instructions about antibiotic use.

Differences in human behavior and attitudes is one example of how adhering to policy guidelines was not always straightforward. At the end of 2018, 19.1% of the Swedish population were foreign-born [17]. PC clinics may be the first point of contact for individuals seeking treatment for infections for which they were commonly prescribed antibiotics in their home country, but not in Sweden. Furthermore, approximately 18% of physicians working in Sweden have earned their original medical degree abroad [18], and many work in PC clinics. There was an impression among some informants that some of these physicians may not yet have accepted expectations to be restrictive when prescribing antibiotics. These are delicate problems and challenges to deal with in efforts to contain antibiotic resistance, as it takes time to change attitudes of both physician and patients regarding antibiotic use. A study on knowledge and attitudes towards antibiotic use and antibiotic resistance in the Swedish population reached the conclusion that actions to improve knowledge should target groups who have misconceptions about antibiotic use and antibiotic resistance, or people with lower levels of education [19]. Our findings suggest that future efforts should also take into account the ever-increasing diversity of the populations in Sweden [20].

Adhering to policy guidelines regarding the use of antibiotics was also not so straightforward, and could even be a dilemma for physicians in both primary and hospital specialist healthcare levels. This is in accordance with a Swedish study on general practitioners’ perceptions of infectious disease management in primary care, which revealed the conflict that could be experienced between wanting to preserve the effectiveness of antibiotics for the future, and helping suffering patients [21]. PC physician informants in our study were also concerned about the long term consequences of antibiotics on intestinal flora. The hospital specialist informants in our study defended the use of antibiotics in hospitals. They focused on managing patients’ immediate clinical needs rather than the consequences of antibiotic overuse. This behavior is congruent with a recent American survey of 400 generalist physicians and 429 infectious disease specialists, who also focused on providing the “newest and best” treatment available for each individual patient, instead of considering the societal risk of antibiotic resistance [22]. This choice can be looked upon as an example of the ‘tragedy of the common’, where the goals of the individual conflict with the goals of society [23]. There is also an ethical perspective to this dilemma for physicians, when acting according to basic ethical principles of beneficence, ‘doing good’ can conflict with ‘non-maleficence, ‘first do no harm’ [24].

Policymaking informants’ observations of how younger doctors dared not question older doctors’ attitudes to antibiotic use point to another challenge yet to overcome, the issue of power in the medical hierarchy in hospitals. Students, during their medical training, learn to accept this hierarchical structure, and their place in it [25]. New in hospitals, fresh doctors can fear judgment of incompetence from senior staff, or dare not challenge or question the judgment of their seniors [25].

In view of the above, and, as stated by policymaking informants, there was a need for continual behavior change regarding antibiotic prescribing in hospitals. For decades, antimicrobial stewardship programs in hospitals have focused on ensuring proper use of antimicrobials to improve patient care, reduce hospital costs, lessen the risk of adverse effects, and reduce or stabilize resistance levels [26]. More emphasis on mitigating antibiotic resistance in these programs may be one way to deal with the dilemma physicians face when trying to reduce AB use in hospitals [26]. However, more emphasis on guidelines may not be enough. A recent study on antibiotic stewardship interventions showed how changes in prescribing practice were facilitated by allowing physicians to be actively involved in discussions about their own prescribing behavior, and in agreeing on specific targets for change in prescribing practice [27].

Stress was perceived to be a hinder to antibiotic resistance work in both hospitals and primary care. How physicians perceive the stressful work environment, high patient volumes, inadequate staffing, bed shortages, or fatigue in Swedish hospitals has been documented in recent studies [28, 29]. As observed in these studies, these factors affected patient safety. According to our informants, and previous research as well [30], these situations could result in not being able to practice restrictive antibiotic prescribing. This intricate relationship between work environment, patient safety, and antibiotic resistance is all the more reason for future healthcare policies to focus even more on improving healthcare work environment.

Most informants, including policymaking informants, appeared to be concerned about resources, the funding of work to contain antibiotic resistance. In our study, policymaking informants seemed to be well informed about problem areas in both primary and hospital care. Their extensive knowledge of antibiotic resistance, and of international collaborative efforts was based on actual experience. In this context, they were experts in their fields. Their concerns about not enough resources and financing of antibiotic resistance efforts was interesting, and may reflect their dedication to antibiotic resistance work. On the other hand, it is possible they indicate the limitation of their position, that of being looked upon solely as experts, and implementers of policies. If this is the case, one can wonder to what extent they are able to influence changes, for example, point to areas in need of improvement.

Many of the informants were not aware of the ‘One Health’ concept. This appears to be one area where governmental expectations described in the introduction have not been met. However, this has been observed among healthcare workers in other countries as well, who did not find ‘One Health’ to be relevant in their daily practice of medicine [31]. This is concerning. Without interest and engagement from physicians and nurses in all healthcare levels, the potential of One Health cannot be realized in Sweden, and globally [31]. Lack of relevance in daily practice may also explain informants’ limited knowledge about antibiotic use and antibiotic resistance in animal food production and the environment.

Based on the discussion above, we suggest that the sense of relative success of policies to contain antibiotic resistance in Sweden so far fits well into McConnell’s description of ‘resilient success’ [32], in that the government appears to be “achieving its’ policy in broad terms”, and “shortfalls, although not insignificant, do not undermine core achievements”.

### Methodological considerations

To the best of our knowledge, this is the first qualitative study that describes how healthcare providers in different levels of Swedish healthcare perceive their efforts to contain antibiotic resistance. As with all qualitative studies, informants’ realities can be interpreted in various ways, and can depend on the researcher’s subjective interpretation. An important issue in qualitative content analysis therefore is achievement of trustworthiness [11]. Four widely used criteria in the discussion of trustworthiness of qualitative studies are credibility, dependability, confirmability, and transferability [11].

For practical and financial reasons, our informants were recruited from, and interviews were conducted in four cities in middle and southern Sweden. In striving for credibility, informants from different levels of healthcare were strategically chosen, so as to increase knowledge of efforts to contain antibiotic resistance from these perspectives. In striving for dependability, the research process was described in detail to make it possible, first, for the reader to understand the logic of the findings, and secondly, to enable a future researcher to repeat the process. To assure confirmability, citations were used to show that the results were grounded in the informants’ experiences and perceptions. Our findings are based on the perceptions of a small sample size of informants from each healthcare level, and cannot be generalized. While there is a possibility the findings can be transferred to other healthcare settings in Sweden, the Swedish context can be a hinder regarding the extent to which they can be transferred to settings or groups outside the country. The overall goal was to gain knowledge of and describe the everyday practice of policy implementation. Data saturation was not our primary focus. Interviews with our strategic sample of informants, although few in number, resulted in an abundance of data, which gave valuable real-life glimpses of how national policies can be translated into daily practice. And, as we note in our discussion, the findings are very much in keeping with previous research. The research team was comprised of researchers with different healthcare backgrounds, pharmacy, medicine, and dentistry. Discussions during the process of the study and drafting of the manuscript revealed these different perspectives and assumptions, which contributed to a deeper understanding of the data, and improving the rigor and quality of the study.

## Conclusions

This study has given in-depth insight into how a group of healthcare policymakers and practitioners perceived the everyday practice of containing antibiotic resistance in Sweden. It has revealed their perceptions of successful efforts to combat antibiotic resistance so far, and their perceptions of problem areas in different healthcare levels. Areas to focus on in future policies have been brought to light, most notably more emphasis on attitude and behavior change, and increasing awareness of antibiotic resistance among both healthcare practitioners and patients. Thus far, these areas have not undermined the core achievements of Swedish national policies. Even so, efforts must continue. Antibiotic resistance will most probably remain a challenge for a long time to come, in Sweden, and the rest of the world.

## Supporting information

S1 Checklist ISSM COREQ checklist. (PDF)

S1 Data Interview guide in English and Swedish. (DOCX)
