# Supplementary material for: The challenges of implementing national policies to contain antibiotic resistance in Swedish healthcare—A qualitative study of perceptions among healthcare professionals
Source: PLoS One. 2020 May 20;15(5):e0233236. doi: 10.1371/journal.pone.0233236 (PMC7239472; doi:10.1371/journal.pone.0233236)
Supplement: S1 Data — (DOCX) [file pone.0233236.s002.docx]

**Supporting information S1**

**Interview guide English**

**Coordination, Accountability, Resourcing, Regulating and Ownership of ABR: A One Health System and Policy Approach (ABR- CARRO)**

**Draft Interview Guide for government level and health care professionals**

**Policy makers/government level**

1. What do you understand by antibiotic resistance?
   1. Probing: Is ABR a problem? If so, where and why?
   2. What do you think are the consequences of antibiotic resistance?
2. What is your role in antibiotic resistance containment?
3. What is your view on the ABR containment?
   1. governance (coordination, accountability)
   2. resourcing
   3. regulation
   4. ownership
   5. implementation
   6. monitoring and evaluation
   7. human, animal and environment
4. What do you understand by one health?

Probe:

- 1. Human, animal and environment

1. What do you think needs to be done to prevent and contain ABR?

Probe:

- 1. Vaccination, infection prevention, bio security, waste disposal, water treatment….

1. What do you think are the barriers & enablers to ABR containment?

**Professionals**

1. What do you understand by antibiotic resistance?

Probing:

- 1. Is ABR a problem? If so, where and why?
  2. What do you think are the consequences of antibiotic resistance?

1. How do you see antibiotic use in humans and animals, and other areas?

Probe:

- 1. Community vs. hospital use in humans, use in viral infections,
  2. Probe growth promoters, prophylaxis and metaphylaxis in (food) animals.
  3. Probe use in agriculture (???).
  4. Disposal? Residues in environment?

1. What is your view on the ABR containment?
   1. governance (coordination, accountability)
   2. resourcing
   3. regulation
   4. ownership
   5. implementation
   6. monitoring and evaluation
   7. human, animal and environment
2. What do you think causes antibiotic resistance?

Probe

- 1. Indiscriminate use (overuse, underuse, misuse)
  2. Use as growth promoters. , prophylaxis and metaphylaxis in (food) animals.
  3. Spread along the food chain.

1. What do you think causes antibiotic resistance to spread?

Probe:

- 1. Infection prevention and control in human health.
  2. Biosecurity in (food) animal health
  3. Use of manure-based fertilizers, water contamination from disposal practices or sub-optimal water treatment.

1. What do you think is your role in preventing/containing antibiotic resistance?

**Interview guide Swedish**

**Coordination, Accountability, Resourcing, Regulating and Ownership of ABR: A One Health System and Policy Approach (ABR- CARRO)**

**Intervjuguide för nyckelpersoner på nationell nivå, samt yrkeskår**

**Nyckelpersoner nationell nivå**

1. Vad betyder antibiotikaresistens (ABR) för dig?

Probe:

- 1. Är ARB ett problem? Om ja, för vem? Var? varför?
  2. Hur ser du på konsekvenserna av antibiotikaresistens?

1. Vad är din roll i kontrollen/begränsningen (”containment”) av ABR?
2. Hur ser du på möjligheten till begränsning av/motverka uppkomst och spridning av ABR?
   1. styrning (koordination, ansvar)
   2. resurser
   3. reglering
   4. ägarskap
   5. implementering
   6. uppföljning och utvärdering
   7. human-, djur- och miljöaspekt
3. Känner du till begreppet “one health”? Hur skulle du definiera begreppet?

Probe:

- 1. human-, djur- och miljöaspekt

1. Vad tror du måste göras för att motverka uppkomst och spridning av ABR?

Probe:

- 1. Vaccination, förebyggande åtgärder, biosäkerhet, avfallshantering, vattenbehandling…

1. Vad tror du är de viktigaste hindren och underlättande faktorerna för att motverka uppkomst och spridning av ABR?
2. Något du vill lägga till?

**Personer ur yrkeskåren**

1. Vad betyder antibiotikaresistens (ABR) för dig?
   1. Är ARB ett problem? Om ja, var och varför? I din egen verksamhet? På annat håll?
   2. Vad tror du är konsekvenserna av antibiotikaresistens?
2. Hur ser du på antibiotikaanvändning för bruk hos människa, djur och andra områden?

Probe:

- 1. Samhälle vs. Sjukhus för humant bruk, användning för viroser etc.
  2. Som tillväxt, profylax och metafylax i djurhållning
  3. I jordbruk?
  4. Avfall? Rester i miljö?

1. Hur ser du på möjligheten till begränsning av/motverka uppkomst och spridning av ABR?

styrning (koordination, ansvar)
